# The impact of socioeconomic and phenotypic traits on self-perception of ethnicity in Latin America

**DOI:** 10.1038/s41598-021-92061-x

**Published:** 2021-06-16

**Authors:** Carolina Paschetta, Soledad de Azevedo, Virginia Ramallo, Celia Cintas, Orlando Pérez, Pablo Navarro, Lucas Bandieri, Mirsha Quinto Sánchez, Kaustubh Adhikari, M. Catira Bortolini, Giovanni Poletti Ferrara, Carla Gallo, Gabriel Bedoya, Francisco Rothhammer, Victor Acuña Alonzo, Andrés Ruiz-Linares, Rolando González-José

**Affiliations:** 1 Instituto Patagónico de Ciencias Sociales y Humanas-CONICET, Puerto Madryn, Chubut Argentina; 2 IBM Research Africa, Nairobi, Kenya; 3 Ciencia Forense, Facultad de Medicina, Universidad Nacional Autónoma de México, Ciudad de Mexico, Mexico; 4 School of Mathematics and Statistics, Faculty of Science, Technology, Engineering and Mathematics, The Open University, Milton Keynes, United Kingdom; 5 Department of Genetics, Evolution and Environment, and UCL Genetics Institute, University College London, London, WC1E 6BT United Kingdom; 6 Departamento de Genética, Universidade Federal do Rio Grande do Sul, Porto Alegre, Brazil; 7 Laboratorios de Investigación y Desarrollo, Facultad de Ciencias y Filosofía, Universidad Peruana Cayetano Heredia, Lima, Peru; 8 Grupo de Genética Molecular (GENMOL), Universidad de Antioquia, Medellín, Colombia; 9 Instituto de Alta Investigación Universidad de Tarapacá, Programa de Genética Humana, ICBM Facultad de Medicina, Universidad de Chile, Santiago, Chile; 10 Escuela Nacional de Antropología e Historia, UNAM, Ciudad de Mexico, Mexico; 11 UMR 7268 ADES, CNRS, Aix-Marseille Université, EFS, Faculté de Médecine Timone, 13005 Marseille, France; 12 Ministry of Education Key Laboratory of Contemporary Anthropology and Collaborative Innovation Center of Genetics and Development, School of Life Sciences and Human Phenome Institute, Fudan University, Yangpu District, Shanghai, 200433 China; 13 Centro Nacional Patagónico-CONICET, Bvd. Brown 2915. U9120ACD, Puerto Madryn, Argentina

**Keywords:** Genetics, Medical research

## Abstract

Self-perception of ethnicity is a complex social trait shaped by both, biological and non-biological factors. We developed a comprehensive analysis of ethnic self-perception (ESP) on a large sample of Latin American mestizos from five countries, differing in age, socio-economic and education context, external phenotypic attributes and genetic background. We measured the correlation of ESP against genomic ancestry, and the influence of physical appearance, socio-economic context, and education on the distortion observed between both. Here we show that genomic ancestry is correlated to aspects of physical appearance, which in turn affect the individual ethnic self-perceived ancestry. Also, we observe that, besides the significant correlation among genomic ancestry and ESP, specific physical or socio-economic attributes have a strong impact on self-perception. In addition, the distortion among ESP and genomic ancestry differs across age ranks/countries, probably suggesting the underlying effect of past public policies regarding identity. Our results indicate that individuals’ own ideas about its origins should be taken with caution, especially in aspects of modern life, including access to work, social policies, and public health key decisions such as drug administration, therapy design, and clinical trials, among others.

## Introduction

Latin American societies exhibit extensive geographic variation in genetic ancestry, reflecting the heterogeneous political and demographic history of the region. In fact, recent genome-wide analyses elucidated the large genetic and cultural geographic variation seen across Latin America, as well as its unique history shaped by the admixture of Native Americans, Europeans and Sub-Saharan Africans^1,2^. Such studies also have shed light on the complexity of the successive admixture events that took place across Latin America, which in turn gave place to the fine-grained genetic sub-structure that characterizes their cosmopolitan population. Since the emancipation wars, Latin American nations implemented different approaches to immigrant integration throughout education, social and cultural policies of ethnicity, race and nation^3–6^. Identity in general, and ethnic self-perception in particular, are dynamic sociocultural phenomena that, at the individual level, are shaped by complex interactions among several factors such as education, physical appearance, family history, and socio-economic context, among others. Therefore, it is expectable that the relationship among genomic ancestry and ethnic self-perception will be not linear, with both, biological and non-biological factors affecting self-perceived ethnicity. The pattern and magnitude of the potential bias between both is of potential interest to many aspects of daily life. For instance, ethnicity classifications are usually used as a variable in social and biomedical research^7–11^. Before moving forward, it is important to define three different concepts: genomic ancestry, ethnicity and race. Genomic ancestry can be seen as the subset of paths through it by which the material in the genome of an individual has been inherited. Because parents transmit only half their DNA to offspring each generation, an individual’s genetic ancestry involves only a small proportion of all their genealogical ancestors^12^. Since chromosomes recombine during the meiosis, it is very common that different positions in the genome have different paths of inheritance^13^.

Regarding “race” and “ethnicity”, several definitions can be invoked. We follow here the conceptualizations made by Roth and Ivermak^14^, who define race as a cognitive structure that divides people into inherent categories, based on phenotypic characteristics, and Cornell and Hartmann^15^ and Roth^16^ who define ethnicity also as a cognitive structure that divides people on the basis of common ancestry, shared history and cultural heritage. Regarding the using of “ethnic self-perception” in the present research, it is important to note that we focus on how individuals estimates their own ancestry, rather than how people identifies themselves with a particular ethnic label or category (e.g. black, mestizo, white, etc.), which is perhaps a more widespread definition. Both concepts overlap to some extent, and some argue that race can be seen as a subtype of ethnicity^17,18^. Moreover, even when the biological concept of “race” cannot be applied to our species^19–22^, race as a social artifact is applicable to a broad range of public policies and can affect concrete individual and group socioeconomic conditions and/or rights. These include the likelihood of accessing educational opportunities (e.g. grants), professional status, neighborhood of residence, or the clinical/research practice of labelling patients or controls with increased or decreased risk of developing specific medical conditions, among others^26,10,11,23–28^.

Unfortunately, research on how ethnic perception establishes and evolves at both individual and community or social levels in modern societies is seriously limited by the scarcity of integrative databases comprising genetic, phenotypic, socio-cultural and socio-economic data. Departing from the comprehensive approach of the Consortium for the Analysis of the Diversity and Evolution of Latin Americans-CANDELA database^1,2,29–33^, here we analyze the relationship between genomic ancestry and ESP, as well as its among-country variation and its putative biases caused by both, biological and non-biological factors. Specifically, we use multivariate and multifactorial analyses to explore the combined effect and the relative weight of variables differing in scale and nature, such as socio-economic status and skin pigmentation, among others. We discuss our results in the light of past and present public policies and its effects on several aspects of daily life in Latin American developing nations.

## Results and discussion

Here we studied a sample of 6094 adult volunteers from Brazil, Chile, Colombia, Mexico, and Peru included in the CANDELA survey (Table 1 and Supp. Table S1). Recruitment was carried out mainly in five locations: Ciudad de México (Mexico), Medellín (Colombia), Lima (Peru), Arica (Chile), and Porto Alegre (Brazil). Phenotypic data, socio-economic information, self-perceived ancestry and DNA samples were collected from each volunteer according to protocols described in ^1,2,29–33^.

**Table 1:** Sample composition, size, countries, sex and birth decade.

Country	Sex	Birth decade				Subtotal
		1990	1980	1970	< 1960	
Brazil	Female	235	496	147	157	1035
	Male	124	216	64	94	498
Chile	Female	128	239	76	70	513
	Male	175	565	132	118	990
Colombia	Female	214	318	46	2	580
	Male	121	241	56	5	423
Mexico	Female	267	422	114	179	982
	Male	139	297	88	103	627
Peru	Female	162	91	16	1	270
	Male	97	56	19	4	176
Subtotal		1662	2941	758	733	6094

### Deviance between ethnic self-perception (ESP) and genomic ancestry.

**Figure 1:**
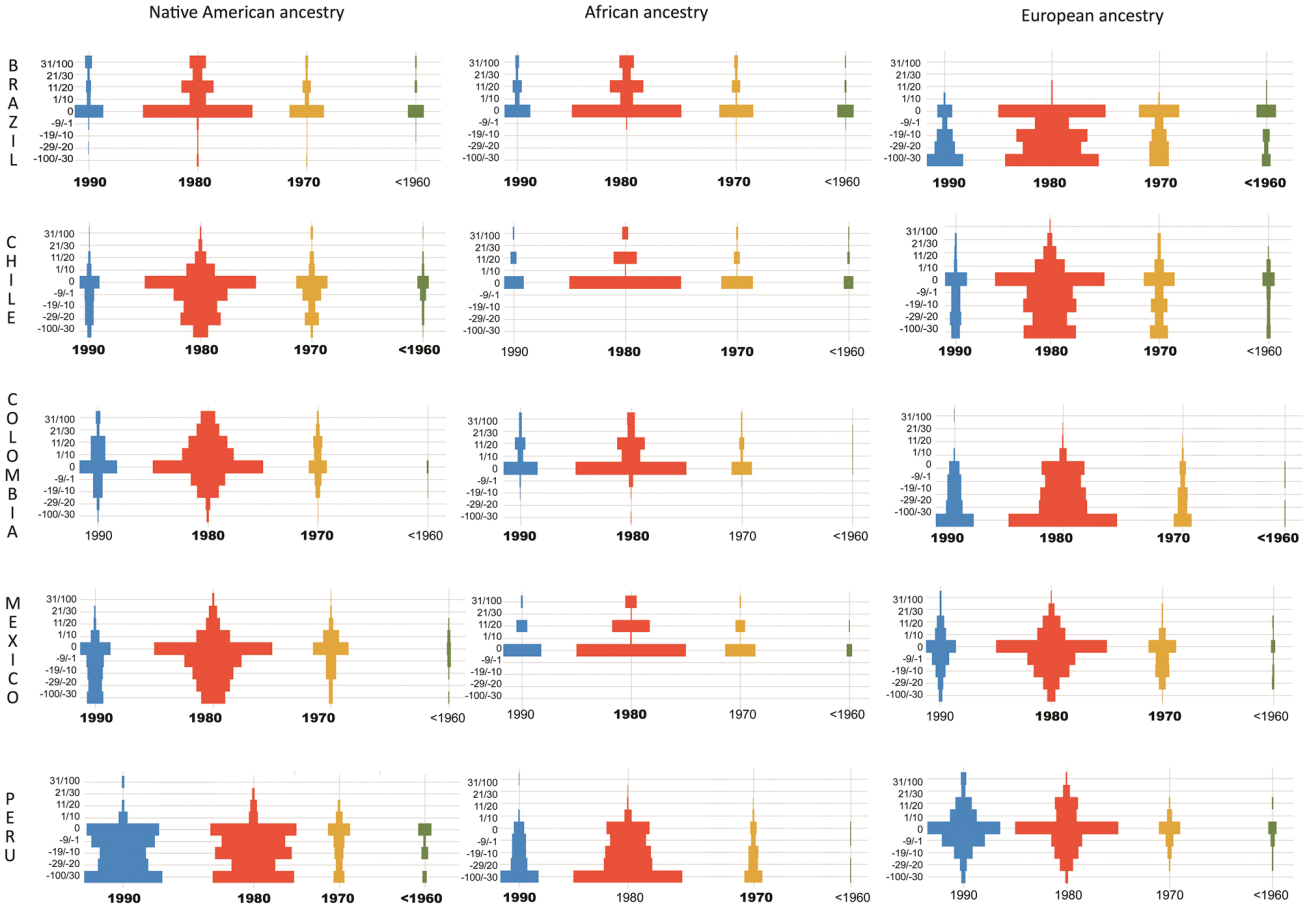
Vertical histograms (pyramid plots) showing the distribution of Delta (Δ) between ESP and genomic ancestry (y-axis) and birth decade (y-axis) separately for ancestry and country. If the percentage of genomic ancestry falls within a given ESP interval, then we considered the case as zero bias. If the bias is positive means that ESP exceeds the genomic estimate, whereas a negative result indicates that ESP is lower than its genomic counterpart. Birth decade in black show significant result to Wilcoxon signed-rank test.

We first approached the distortion or difference (Delta) between ESP and genomic ancestry by computing the subtraction between both parameters. Vertical histograms depicting the Delta (Δ) values of ESP to genomic ancestry for each country are shown in Fig. 1. As observed, the greatest amount of individuals falls within the “zero” bar, thus indicating that most individuals present a non-biased self-perception regarding their ancestry (Δ = 0). Nevertheless, there are interesting deviations that deserve further analysis. In Colombia and Brazil, for instance, for any age-category, European ancestry is recurrently underestimated (e.g. self-perception of European ethnicity is lower than European genomic ancestry). Conversely, young-adults from Peru (those born in the 80’s) tended to underestimate their Native American ancestry. In Brazil, however, Native American ancestry is overestimated. In general, we report here a general trend to overestimate African ancestry (intended here as ethnicity), along with a general underestimation of European ancestry.

Our results are congruent with previous studies that report non-linearity among both variables, possibly due to environmental/socio-economic traits affecting the perception of phenotypic variation, and to the genetic architecture of physical appearance traits which probably induce self-perception. Parra and collaborators^34^, for instance, focused on African ancestry and physical appearance as a proxy to self-perceived African ethnicity on samples of rural and urban individuals from Brazil. Their results indicated that, at an individual level, skin pigmentation is a poor predictor of genomic African ancestry estimated by molecular markers. Interestingly, Ventura Santos and collaborators^11^ introduced the question on how social debates about race, science, and society, or the formulation of public policies designed to address these questions, can operate as factors conditioning the individual self-perception of race and ethnicity. Based on an extensive survey on Rio de Janeiro students and their individual approaches to self-classification, the authors demonstrated that self-classification can vary across short intervals of times (e.g. four months), which indicates how large can be the influence of context and the intrinsic malleability of this trait.

On a recent study made on three Brazilian cohorts, Lima Costa and collaborators^35^ showed that self-classification is not random with respect to genome individual ancestry, and detected some tendency to whitening ethno-racial self-identification in persons from Salvador da Bahia, where African ancestry is more frequent. However, such a trend was not observed on the remaining two cohorts, where European ancestry predominates^35^. In concordance, Telles and Paschel^36^ finds reflect a rapidly changing political and social context in Colombia and Brazil, as a consequence of black movements creating counter-narratives that change nation-centered initiatives that have promoted whitening, towards stronger black identity. The state is not the only who shapes racial schemas and its concomitants racial classification and identification. In fact, whereas states can promote whitening and expanding the boundaries around “whiteness”, black social movements may be counteracting this trend by expanding the boundaries around “blackness”. These movements not only have they pressed the state to adopt multicultural and antiracism legislation, but also have encouraged people of African origin to identify as black, challenging racist discourses^37–39^. Thus, changes in state policies, nationalist narratives, and social movement actions could shift national racial schemas and classification systems, and countries like Colombia and Brazil are examples of such processes.

On a previous study made on the CANDELA sample, we showed that genetic ancestry impacts many aspects of physical appearance, which in turn influences ESP but also biases it relative to genetically estimated ancestry^2^. Results presented here also confirmed that highly admixed people and Native American descendants present lower deviations between perceived ethnicity and genomic ancestries.

### Deviance between ESP and genomic ancestry across age ranks/countries.

The results concerning the Wilcoxon signed-rank and Monte Carlo test are incorporated in Fig. 1, where those decades that showed significant differences between ESP and genomic ancestry are shown in bold, and are also presented in more detail in Supp. Table S2. Significant differences between ESP and genomic ancestry are observed across the majority of comparisons. When observed from the age-rank perspective, people born in the 60’s or before tend to exhibit greater agreement between ethnic perception and genomic ancestry (4 out of 15) in comparison to younger people (10–13 out of 15). When analyzed from the country perspective, Brazil and Chile show greater disagreements between ESP and ancestry (10–9 out of 12), with Colombia showing an intermediate position (8 out of 12), and Peru and Mexico exhibiting a slightly superior congruence between both parameters (6 out of 12). Self-perception of Amerindian ethnicity exhibit stronger distortion in relation to genomic ancestry (16 out of 20), whereas African and European self-perceived ethnicities are more congruent with their congruent genomic counterparts (10–13 out of 20).

**Figure 2﻿:**
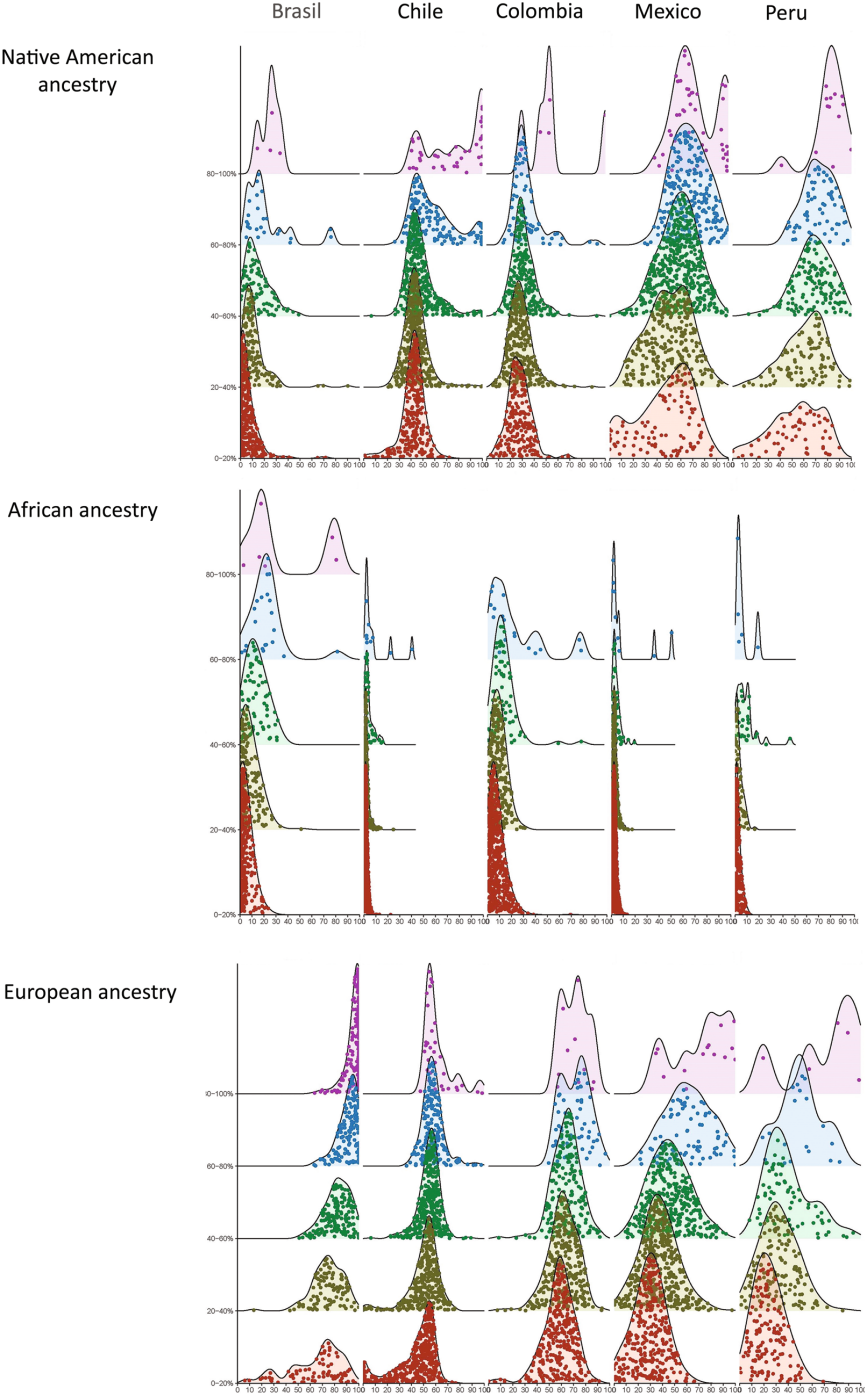
Modal distribution of individuals for the ESP (y-axis) versus genomic ancestry (x-axis) separately for each country and ancestry. The fine grained distribution of individual on the ESP versus genomic ancestry space for each country/ancestry segmentation of the sample. Every point represent a value of an individual.

Modal distribution graphs presented in Fig. 2 help to interpret some specific deviations from the expected normal distribution in different country/ethnicity comparisons. Under this approach, a non-biased distribution (e.g. no differences between ESP and genomic ancestry) will be represented as a bell curve distributed around the mean of ESP declared. For instance, in the case of the 60–80% of ESP for a given ancestry, the center of the bell will be located around 70. Thus, in one hand, some distortions can be defined as a simple deviation of the entire distribution bell in the direction of over or under estimation of specific genomic ancestries, as is the case of the overestimation of Native ancestry in the Brazilian sample. On the other hand, distortions cannot be defined as easily, as is the case of people declaring low self-perception of Native ethnicity in México, or European ethnicity in Brazil, whose genomic ancestry corresponds to a wide range of distribution, that is low, intermediate and high ancestry. In combination, the Wilcoxon signed-rank and the modal distribution results suggest some complex phenomena underlying the observed distortions involving specific age ranks, ancestries, and countries. Even when variation in the distortion between ESP and genomic ancestry across age ranks do not respond to a common across-country pattern, with some countries showing under or over estimation of a given parental population, our results show that people born in the 60’s or before exhibit lower distortions when compared to younger people. Also, they can be interpreted in the light of the increasing importance of debates about ethnicity and race that many Latin American countries experienced during the last decades, with a kind of climax in the 1990s that derived on specific policies implemented especially in the areas of education and health. In Colombia, for instance, the “Constitución Política de 1991” explicitly recognize rights of ethnic minorities and afro-descendants, and consolidates their inclusion into a Political System^40^ which enhances the chances of achieving places of political relevance to African or Native American descendants. On a similar way, Brazil and Peru also introduced political changes relative to ethnic minorities. After 2012, Brazil^41^ implemented social and racial quota at the universities and distributed them between “blacks” or indigenous, according to self-definition. Nevertheless, committees have to validate such assignments based on phenotypic traits, in the case of “blacks” and the link of the indigenous individual with his original village, usually located in reserves. In other words, those people who identify themselves as belonging to any of these groups increased their chances to get a place at the university. In Peru, during the 90 the social exclusion did not allow certain social groups such as African and Native American descendants to participate in formal economic, social, cultural and political spheres. Consequently, these groups developed alternative strategies in recent times, aimed to reinforce their ethnic identity. In the case of Native American descendants, the strategy consisted on a double process of assimilation and cultural resistance. The Andean population, for instance, which constituted the main core of rural migrants arriving to suburban spaces in large cities opted for the abandonment of some ethnic markers (mainly clothing and language), but maintained a core of customs and own values. Regarding Afro-Peruvian groups, Benavides and collaborators^42^ reported that they developed a kind of pride "Black" focused on his "race", which helped to “racialize” their own identification group.

Besides the potential effect of contemporary policies, implemented by constitutional nations in Latin America during the second half of the XXth century, it is noteworthy that some aspects of social organization can be traced back to colonial or early-post colonial epochs. This can be the case of early colonial elite decisions aimed to ‘‘whiten’’ the population through miscegenation rather than impose segregation, with ethno-racial classification left to individual perception^35^. In line with previous studies^35^, our results regarding greater across-ages disagreements in the ancestry-type relationship in Brazil and Chile may indicate a long-term effect of past policies regarding segregation versus integration laws. This is why, perhaps, this consistent distortion among ESP and genomic ancestry behaves as a more stable and long-term effect in Brazil and Chile. In other words, a potential effect of the lack of segregation laws defining who should belong to an ethnoracial group at the very beginning of some cosmopolitan Latin American societies, is perhaps the primary cause of distortion among ESP and genomic ancestry across different age ranks.

### The contribution of different kinds of variables to ESP

Multiple Factorial Analyses (MFA) approaches are useful to attempt a comprehensive picture of the relative importance of phenotypic and socio-cultural factors underlying the individual building of self-perceived identity. MFA provides the advantage that the complex interactions among self-perception, physical factors such as external appearance and socio-cultural traits such as access to formal education and/or welfare can be studied on a single analytical framework, making the unravelling of specific interactions more accessible. To develop such analyses, large and comprehensive databases are needed, with different types of variables measured on each individual.

**Figure 3:**
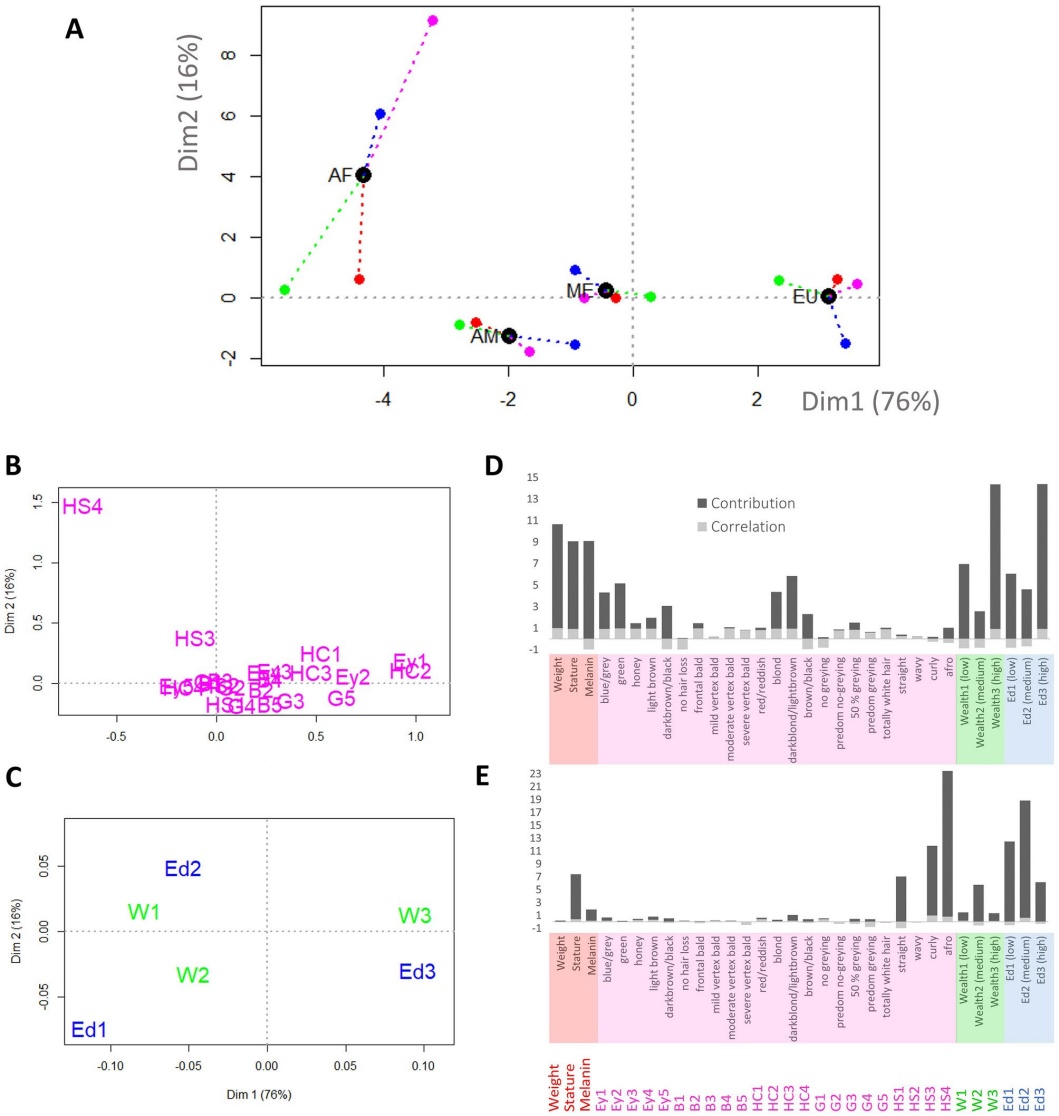
MFA results for the pooled sample (all countries). (**A**) The first two dimensions of the MFA showing simultaneously the global average (black dots) for each ESP category (AF: Africans, AM: Amerindians, EU: Europeans, and ME: mestizos), and the variation among them according to each block of variables (quantitative variables or quanti_PHEN; qualitative variables or quali_PHEN; wealth variables; education variables). Global average (black dots) are joined to their corresponding partial points, depicting the influence of each set of variables (e.g. colored dots or partial points denote the position of each individual seen only by a given group of variables). (**B**, **C**) Plot of MFA’s coordinates of the qualitative phenotypic (**B**) and sociocultural (**C**) categories as listed in Supp. Table S3. (**D**, **E**) Correlation and Contribution values of all individual variables to the MFA’s first (**D**) and second (**E**) dimensions.

Here we used the CANDELA database to explore further questions regarding the determinants of ESP, such as what is the relative weight of very different variables. MFA results are presented in Fig. 3, which shows the distribution of individuals (sample means) for the global analysis (all countries together). The four sub-samples represent averaged individuals regarding its ESP, that is mostly Mestizos, mostly African, European, or Native American. These averaged sub-samples were plotted across the two first dimensions of the MFA space (Fig. 3A) where its relative positions are given by the inertia exerted by each block of variables. Thus the resultant position depends on the balance among the different blocks of variables, and the partial individuals’ superimposed representation depict each sub-population viewed only by a given group of variables. This representation allows exploring, for instance, if the variables of one group provide the same information as the variables of the other groups, or whether there is partly shared information and partly group-specific information. In general, both in the global (Fig. 3A) and the by-country analyses (Supp. Figs. S1–S5), the first dimension of the MFA tend to separate the subsample of European self-perception apart from the Native American and/or African subsamples, with admixed individuals occupying a central position, near the origin of coordinates. Note that the group of self-perceived as mostly African were excluded from the MFAs in the cases of Peru, Mexico and Chile because of its low sample size. In these specific cases, self-perception Mestizos are, again, placed near the origin of coordinates, whereas the first MFA axis separates European from Native American self-perception groups. Specifically, individuals characterized as presenting higher education and wealth standards (Fig. 3C) tend to self-perceive as of European ethnicity, as well as those individuals carrying blue/gray/green eye color, advanced graying, or blonde hair color (Fig. 3B). In the case of self-perceived Europeans, their position on the positive values of the first dimension seems to be mostly explained by hair color, higher education levels and wealth standards (Fig. 3D). Individuals self-perceived as mostly African are placed in the negative space of the first dimension and the positive values of the second one (Fig. 3A). The traits characterizing this specific space are afro hair-shape and lower education and wealth levels (Fig. 3B,C). However, note that the relevance of possessing afro hair shape as a determinant to trigger an African ethnic self-perception seems to be the most remarkable underlying criteria (Fig. 3E). The relationship between lower education level and the African self-perception was previously reported by Telles and collaborators^43^, they found for eight countries (Bolivia, Brazil, Colombia, Dominican Republic, Ecuador, Guatemala, Mexico and Peru) that more pigmented individuals consistently exhibited greater educational penalties, despite remarkable social, political, and historical differences among countries. When all the countries are analyzed together, individuals self-perceived as admixed or as mostly of Amerindian are placed near the coordinates’ origin, with no traits, nor phenotypic neither socio-cultural, mainly influencing their position in the muti-factorial space of self-perceived ethnicity.

Of course, the detailed analysis by each country shows subtle variations on the patterns described above. The most remarkable, perhaps, is the impact of higher education levels on the position of self-perceived Europeans across the first dimension. Whereas it is an important trait forcing the group towards positive values in Colombia (Supp. Fig. S3), Mexico (Supp. Fig. S4) and Peru (Supp. Fig. S5), it has no relevance in Brazil (Supp. Fig. S1), and seem to have the opposite effect in Chile (Supp. Fig. S2) (e.g. triggers the group towards the coordinates’ origin). A similar behavior pattern can be observed on the Amerindian counterpart of the country-specific graphs, where education level behaves very differently depending on the country. Other traits, such as curly and afro hair shape, seems to be equally influential across the three countries where such African sub-sample achieved enough sample size in order to be analyzed.

Our MFA results indicate that the position in the statistical space cannot be extrapolated from one ethnic group to another. In other words, the specific biological and non-biological traits that contribute to the self-perceived ethnicity in one of the studied groups is not informative about the determinants on other groups. It is worth noting that admixed and Native American self-perception groups tend to exhibit lower distortions, a result that suggest that special attention should be provided to scenarios when self-perceived African or European ethnicity can influence on public decision processes. Interestingly, the presence of specific physical attributes such as afro hair is determinant to increase the self-perception of African ethnicity, whatever the values registered in other variables or block of variables. This suggest that the distortion of ESP can fluctuate below some thresholds imposed by the presence of specific attributes that funnel the trait’s expression and variation to some extent.

As many previous studies our results open several questions regarding the using of ethnicity as perceived by ourselves or by others, as a trait of importance in the realm of health practices, work and education policies, etc. Most health researchers and practitioners, for instance, have a concept of ethnicity, whether personal or disciplinary, that they apply when reading or hearing about ethnic differences. Since ethnicity is infrequently defined, its usage in specific circumstances may remain elusive^44^. Our paper suggest that caution is needed when ethnicity self-declaration is used in the daily life and public domain in Latin America. Present times face the challenge of guaranteeing more and better access to vulnerable people to the public health, education and work systems. In this context, future research will be benefited by more fine-grained longitudinal surveys, or genealogically structured composite data that will help to understand how self-perception evolves across generations into a familiar and/or population level. However, there is an urgent need to revisit public policies and clinical practices based on self or other-reported ethnic classifications, which can derive in serious scientific flaws or social inequalities due to the complex, dynamic, and non-linear behavior of this attribute. Certainly, this discussion will be surrounded by many complexities. For instance, there is a major difference between the use of ethnic identifiers for medical purposes, such as diagnosis and treatment, where the actual genomic characteristics of a patient may be medically important and the question is the degree to which ethnic self-identification can be a useful guide to genomic ancestry; and for social policy purposes, where the genomic profile is irrelevant. Also, what (usually) matters in these social policy domains is how other people see an individual in ethnic and racial terms, rather than how an individual sees him/herself. After long debates, some countries have already well recognized that both, self and others-identifications are problematic. Thus, in the light of the complexities involved into the processes that led to self (or others) ethnic identification evidenced by our results and by previous research, workable alternatives including employment, health, and social policies at the supra-individual, community level are needed in order to overcome the limitations discussed above.

## Materials and methods

### Sample

Since 2010 to 2013 CANDELA recruited more than 9000 volunteers from five countries in Latin America. All participants provided written informed consent, and ethics committee's approval was obtained from: Universidad Nacional Autónoma de México (Mexico), Universidad de Antioquia (Colombia), Universidad Peruana Cayetano Heredia (Peru), Universidad de Tarapaca (Chile), Universidade Federal do Rio Grande do Sul (Brazil), and University College London (United Kingdom). All participants provided written informed consent. Blood samples were collected by a certified phlebotomist and DNA extracted following standard laboratory procedures. To preserve the privacy of participants the information associated to each one was anonymized by an identification code. Further details can be consulted in Ruiz-Linares and collaborators^2^. All methods and procedures used here were performed in accordance with relevant guidelines and regulations.

### Genomic ancestry

Blood samples were collected by a certified phlebotomist, and DNA was extracted following standardized protocols^2^. Samples were genotyped using the Illumina OmniExpress array (~ 730K SNPs). The SNPs were pruned to remove Linkage Disequilibrium and 90,000 SNPs were left for analysis after removing correlated, the ancestry estimation was performed with this SNP data. Supervised ancestry estimation using ADMIXTURE was performed, estimating three ancestry components for each individual: Native American, European, and African^1,45^.

### Self-perceived ethnicity

Several socio-cultural traits, including self-perceived ethnicity were obtained through a structured questionnaire applied to each volunteer. The questionnaire included an item exploring self-perception of African, European, and Native American ethnicity proportions. The volunteer response was proposed as a five-point scale, expressed in percentage ranges and in words: (1) 0–20% (none or very low), (2) 20–40% (low), (3) 40–60% (moderate), (4) 60–80% (high), and (5) 80–100% (very high or total). The volunteers selected a range for each self-perception (African, European, Native American). The questionnaire also recorded information on the place of birth of the volunteer.

### Socio economic (SES) covariates

Socio economic status estimators are characterized by its intrinsic complexity in sociological terms, and its multifactorial and nonlinear nature when statistically approached^46,47^. Here we used information of two indicators of socioeconomic position. The first one focuses on the highest formal education level attained, categorized as: (1) none/primary/technical, (2) secondary and (3) university and post-graduate. The second indicator is a wealth index obtained from a list of domestic items/appliances used to assess living standards, such as home ownership, number of bathrooms at the place of residence, ownership of household items (cars, bicycles, fridge, freezer, dishwasher, TVs, radios, CD/DVD players, vacuum cleaner, washing machine) and availability of domestic service. We used Principal Component Analysis to examine the variability of each country sample and retained the first principal component as an indicator of wealth. The wealth variable were divided into three groups: low, medium and high. To allow comparisons across countries we converted an individual’s wealth score to a decile scale within each country^33,45^.

### Statistical methods

Self-perception was recorded as five intervals of 20% (0–20, 20–40, 40–60, 60–80, 80–100), whereas the genomic ancestry estimate is expressed as a percentage on a continuous scale^1,2^. Because self-perception is treated here as an interval, the bias was measured as the distance of the closest boundary of the interval to the genomic ancestry value. The resultant delta values were used to perform country-specific bias analyses, vertical histograms, and to explore any difference among age intervals. If the ESP range includes the genomic ancestry, then Delta is zero (not biased). Conversely, if the ESP is higher than genomic ancestry the result is positive (overestimation), whereas if the ESP is lower than genomic ancestry the result is negative (underestimation).

To evaluate any statistical differences between self-perceived ethnicity and genomic ancestry we computed the non-parametric Wilcoxon signed-rank test. Significance values were obtained using a Monte Carlo resampling procedure^48^.This test is used to compare two matched samples to assess whether their population mean ranks differ.

### Multiple factor analysis (MFA)

The data compiled in the CANDELA database differs, by its own nature, in types and scales, which turns difficult comprehensive analyses of complex phenomena. To overcome this problem we used Multiple Factor Analysis (MFA), a method that analyzes a given set of observations described by several “blocks" or sets of variables that can differ in their nature (e.g. nominal or quantitative)^49,50^. Of course, variables within a given block must belong to the same type (quantitative or categorical) but blocks of variables can vary in nature from one to another. Our interest in this method is due to its being able to analyze a mix data table as a whole, but also its ability to simultaneously compare information provided by various information sources. In fact, MFA can be seen as a type of Principal Component Analysis on a weighted matrix that balances information provided by different groups of variables. Applied to the objective of this paper, MFA is implemented to explore, on a single and integrated way, how different types of variables (external phenotypes, socio-economic status, etc.) influence the relative position of self-perceived ethnicity groups on the multifactorial statistic space. As a first step, we used the individual’s self-perceived ethnicity information to create four “self-perceived categories”: individuals that consider themselves as mainly Native American, mainly African, mainly European, or admixed. For instance, if an individual classifies himself or herself as having 60% or more (60–80% or 80–100%) of any of the major parental ethnic groups, the individual was classified as belonging to that group, otherwise if all possible ethnic groups were valued with less than 60% by the individual (0–20%, 20–40% or 40–60%) he/she was classified as admixed (or mestizo). The resulting four categories were used as dependent variables on a MFA design^49,51^. The variables in our dataset are either quantitative (e.g. melanin values, wealth index, etc.) or qualitative/categorical (e.g. levels of balding, categories of hair shape, level of formal education, etc.), and all these variables describe either physical or socio-cultural phenotypic traits. To run the MFA, we arranged these variables into four blocks of our interest: phenotypic traits measured in a quantitative scale, which were averaged for each self-perception categories (e.g. stature, etc.); phenotypic traits measured in a qualitative scale (e.g. eye color, hair shape, etc.), which were entered to the analysis as frequencies for each self-perception categories; the wealth index described before was arbitrarily partitioned into three levels (low, medium, high) and were entered to the analysis as frequencies of individuals at these levels for each self-perception categories; finally, the information of level of formal education achieved by the individual was also codified as low, medium or high and entered to the analysis as frequencies (see Supp. Table S3). To elucidate how ESP is composed at the group level, we analyzed the relative influence of the different blocks of variables on the position of the four categories (Native Americans/Europeans/Africans, admixed) into the multifactorial space. MFA was run on R Core Team^52^ using the FactoMineR package^53^.

## Supplementary Information

Supplementary Information.
