# Supplementary material for: The impact of socioeconomic and phenotypic traits on self-perception of ethnicity in Latin America
Source: Sci Rep. 2021 Jun 16;11:12617. doi: 10.1038/s41598-021-92061-x (PMC8209281; doi:10.1038/s41598-021-92061-x)
Supplement: Supplementary file 1 — Supplementary Information. [file 41598_2021_92061_MOESM1_ESM.docx]

**Supplementary Information**

**The impact of socioeconomic and phenotypic traits on self-perception of ethnicity in Latin America.**

Carolina Paschetta, Soledad de Azevedo, Virginia Ramallo, Celia Cintas, Orlando Pérez, Pablo Navarro, Lucas Bandiaeri, Mirsha Quinto Sánchez, Kaustubh Adhikari, M. Catira Bortolini, Giovanni Poletti Ferrara, Carla Gallo, Gabriel Bedoya, Francisco Rothhammer, Victor Acuña Alonzo, Andrés Ruiz-Linares, Rolando González-José*.

Paste the full author list here

Rolando González-José

Email: *rolando@cenpat-conicet.gob.ar

**This PDF file includes:**

Figures S1 to S5

Tables S1 to S3


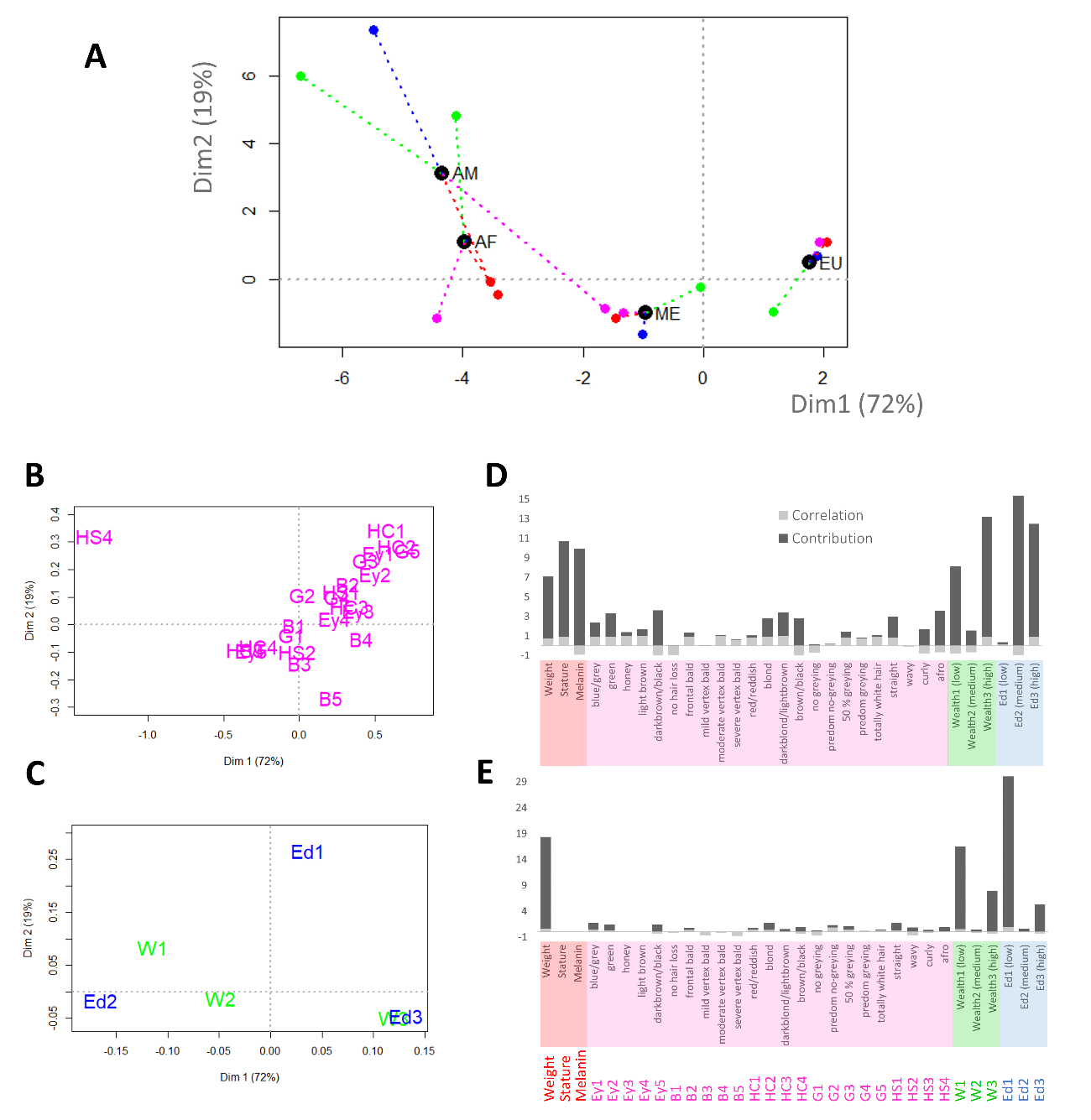


**Figure S1:** **MFA results for Brazil sample.** **A:** The first two dimensions of the MFA showing simultaneously the global average (black dots) for each ESP category (AF: Africans, AM: Amerindians, EU: Europeans, and ME: mestizos), and the magnitude and direction of each block of variables, represented as colored vectors: red, phenotypic traits measured in a quantitative scale; magenta, phenotypic traits measured in a qualitative scale; green, wealth scores; blue, education scores. Global average (black dots) are joined to their corresponding partial points, depicting the influence of each set of variables (e.g. colored dots or partial points denote the position of each individual seen only by a given group of variables). **B, C:** Plot of MFA´s coordinates of the qualitative phenotypic (**B**) and sociocultural (**C**) categories as listed in Supp. Table S3. **D, E:** Correlation and contribution values of all individual variables to the MFA´s first (**D**) and second (**E**) dimensions.

**
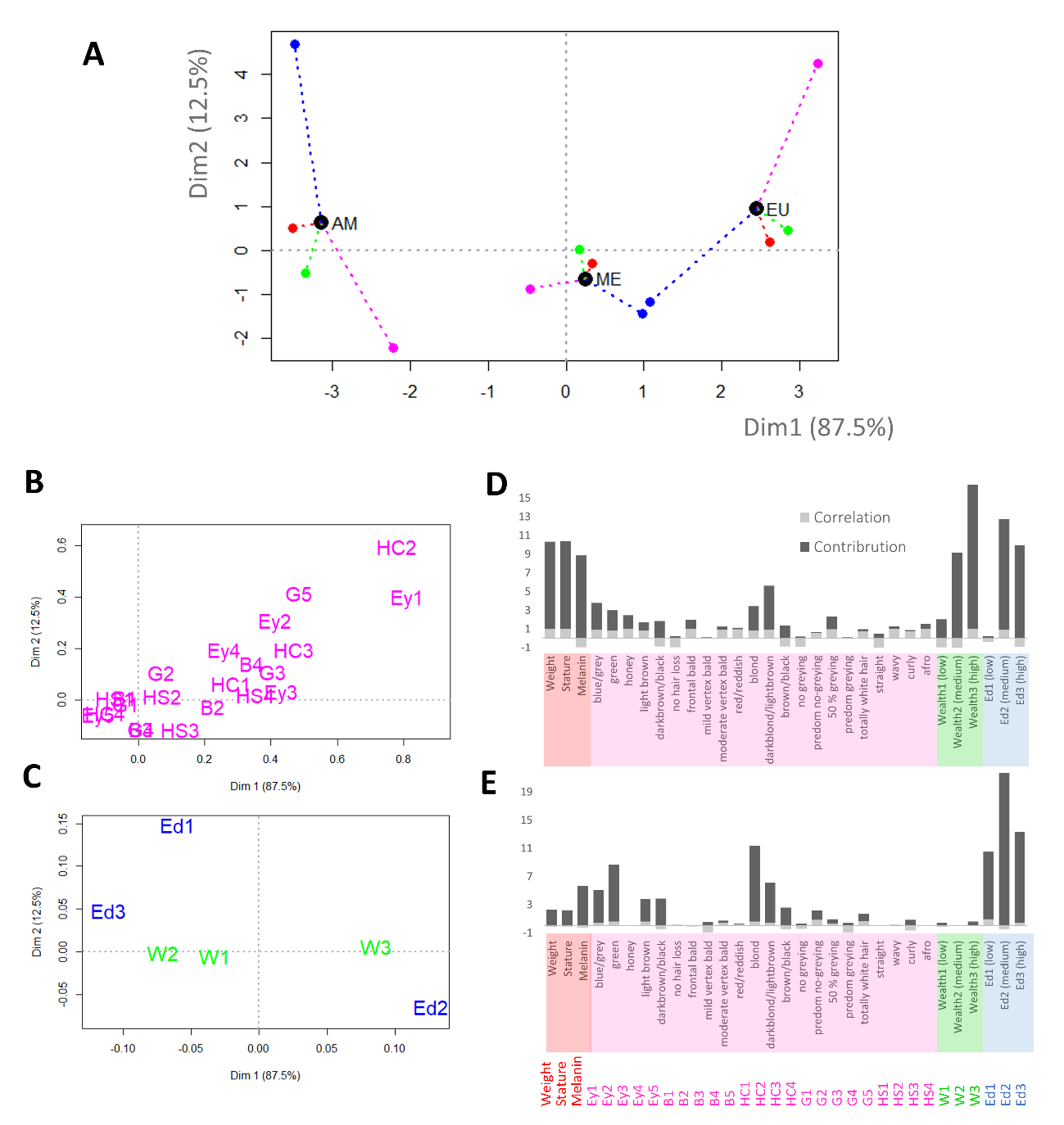
**

**Figure S2:** **MFA results for Chile sample. A:** The first two dimensions of the MFA showing simultaneously the global average (black dots) for each ESP category (AF: Africans, AM: Amerindians, EU: Europeans, and ME: mestizos), and the magnitude and direction of each block of variables, represented as colored vectors: red, phenotypic traits measured in a quantitative scale; magenta, phenotypic traits measured in a qualitative scale; green, wealth scores; blue, education scores. Global average (black dots) are joined to their corresponding partial points, depicting the influence of each set of variables (e.g. colored dots or partial points denote the position of each individual seen only by a given group of variables). **B, C:** Plot of MFA´s coordinates of the qualitative phenotypic (**B**) and sociocultural (**C**) categories as listed in Supp. Table S3. **D, E:** Correlation and contribution values of all individual variables to the MFA´s first (**D**) and second (**E**) dimensions.

**
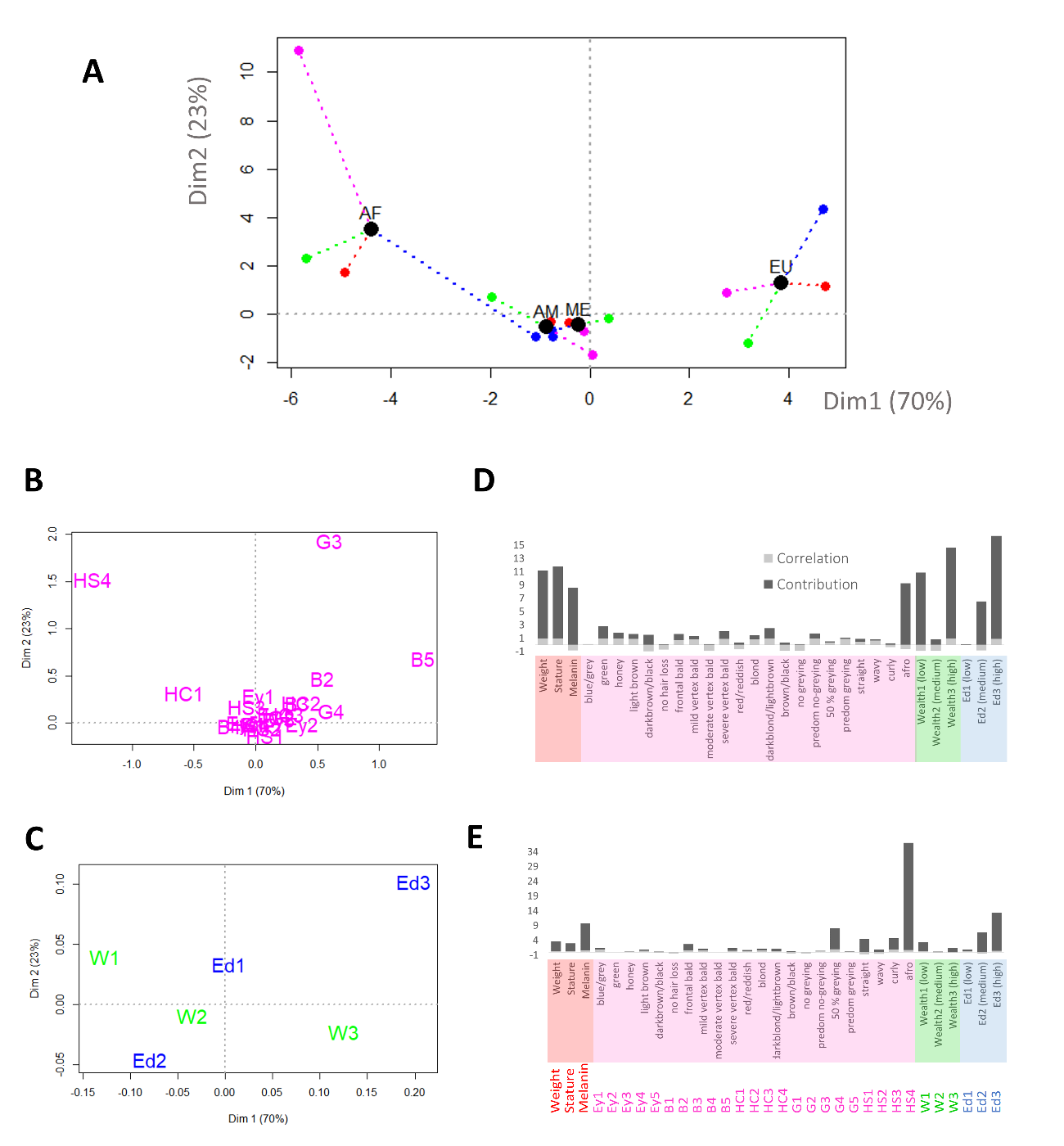
**

**Figure S3:** **MFA results for Colombia sample. A:** The first two dimensions of the MFA showing simultaneously the global average (black dots) for each ESP category (AF: Africans, AM: Amerindians, EU: Europeans, and ME: mestizos), and the magnitude and direction of each block of variables, represented as colored vectors: red, phenotypic traits measured in a quantitative scale; magenta, phenotypic traits measured in a qualitative scale; green, wealth scores; blue, education scores. Global average (black dots) are joined to their corresponding partial points, depicting the influence of each set of variables (e.g. colored dots or partial points denote the position of each individual seen only by a given group of variables). **B, C:** Plot of MFA´s coordinates of the qualitative phenotypic (**B**) and sociocultural (**C**) categories as listed in Supp. Table S3. **D, E:** Correlation and contribution values of all individual variables to the MFA´s first (**D**) and second (**E**) dimensions.

**
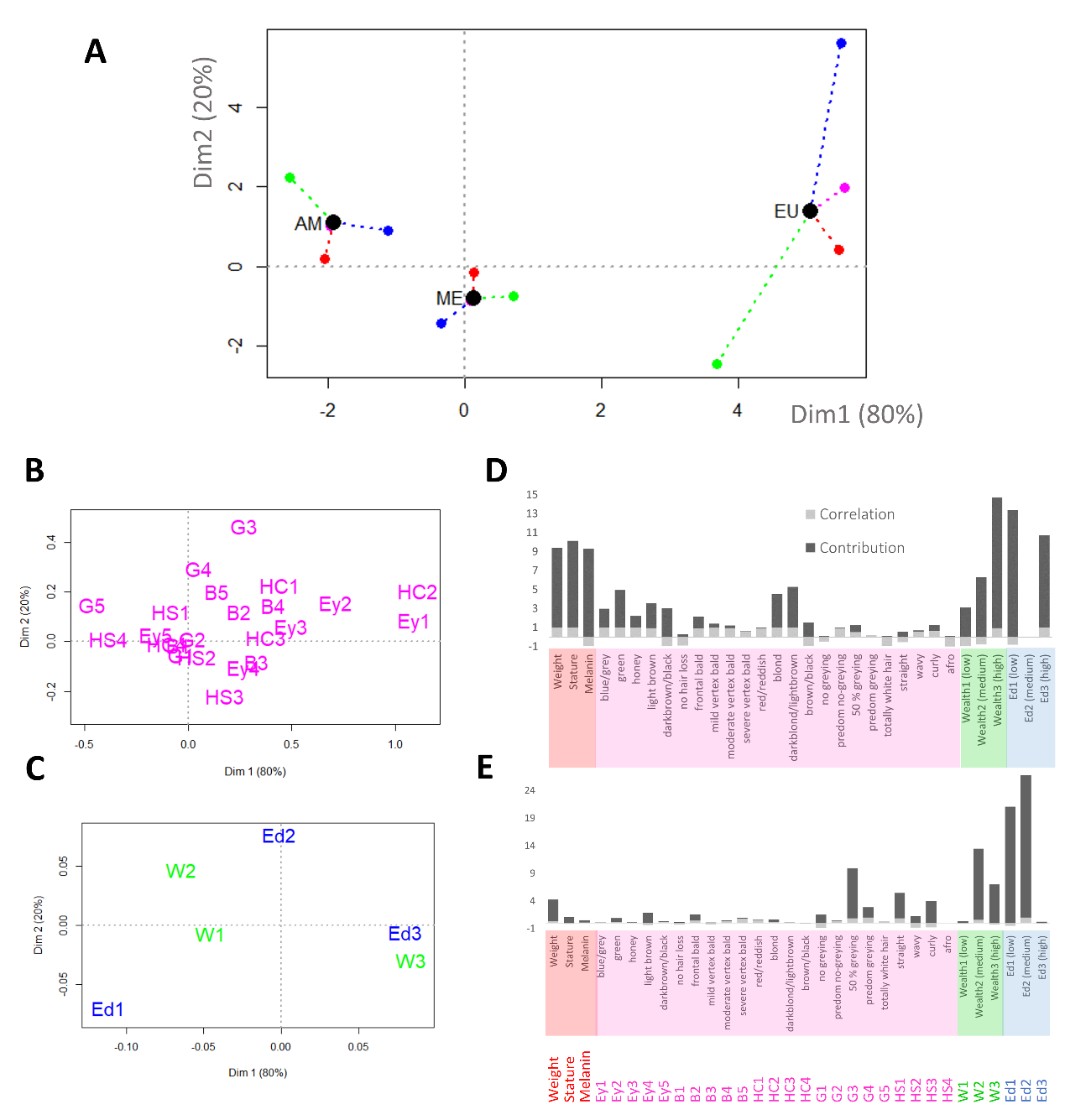
**

**Figure S4:** **MFA results for Mexico sample. A:** The first two dimensions of the MFA showing simultaneously the global average (black dots) for each ESP category (AF: Africans, AM: Amerindians, EU: Europeans, and ME: mestizos), and the magnitude and direction of each block of variables, represented as colored vectors: red, phenotypic traits measured in a quantitative scale; magenta, phenotypic traits measured in a qualitative scale; green, wealth scores; blue, education scores. Global average (black dots) are joined to their corresponding partial points, depicting the influence of each set of variables (e.g. colored dots or partial points denote the position of each individual seen only by a given group of variables). **B, C:** Plot of MFA´s coordinates of the qualitative phenotypic (**B**) and sociocultural (**C**) categories as listed in Supp. Table S3. **D, E:** Correlation and contribution values of all individual variables to the MFA´s first (**D**) and second (**E**) dimensions.

**
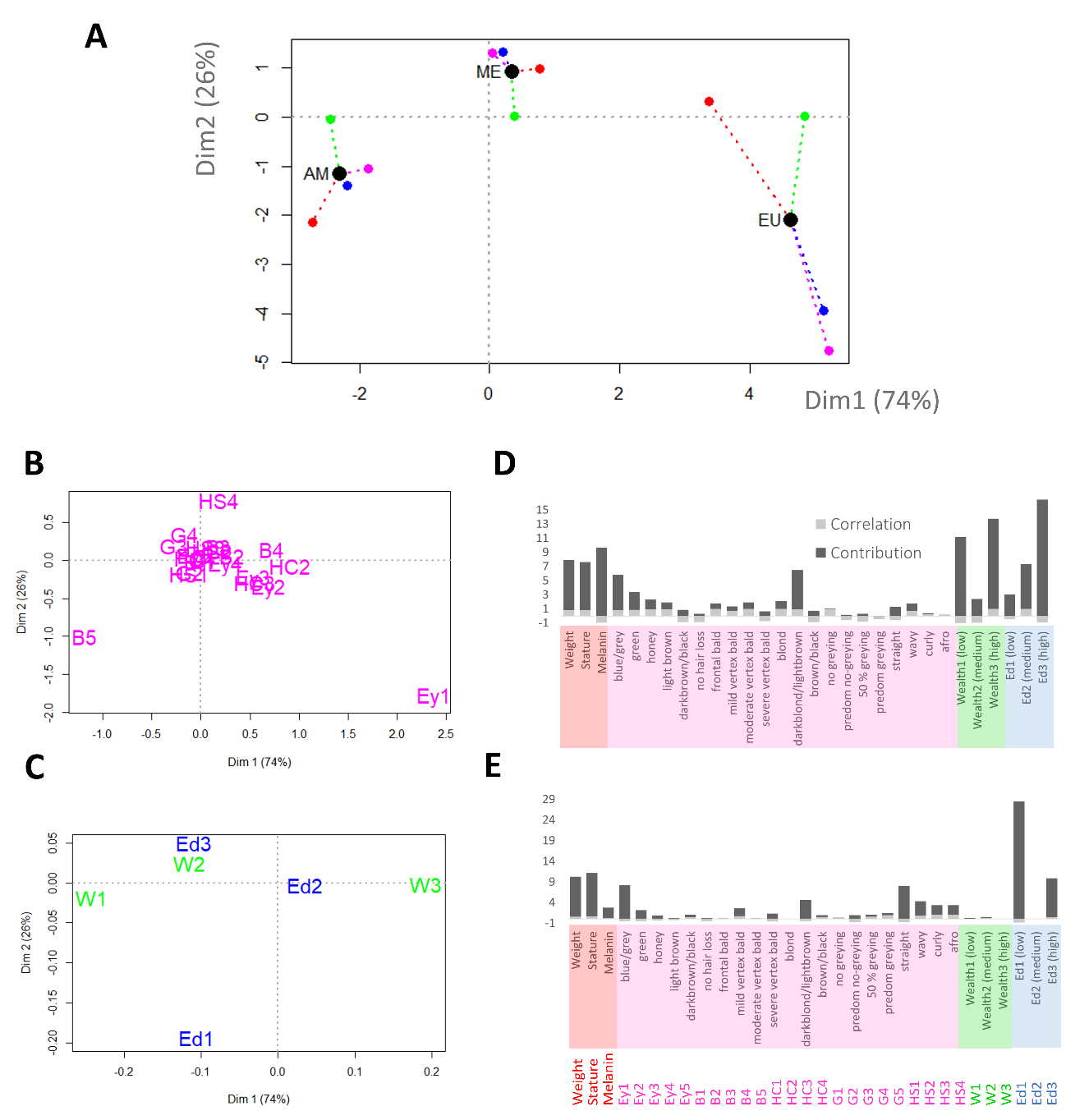
**

**Figure S5:** **MFA results for Peru sample. A:** The first two dimensions of the MFA showing simultaneously the global average (black dots) for each ESP category (AF: Africans, AM: Amerindians, EU: Europeans, and ME: mestizos), and the magnitude and direction of each block of variables, represented as colored vectors: red, phenotypic traits measured in a quantitative scale; magenta, phenotypic traits measured in a qualitative scale; green, wealth scores; blue, education scores. Global average (black dots) are joined to their corresponding partial points, depicting the influence of each set of variables (e.g. colored dots or partial points denote the position of each individual seen only by a given group of variables). **B, C:** Plot of MFA´s coordinates of the qualitative phenotypic (**B**) and sociocultural (**C**) categories as listed in Supp. Table S3. **D, E:** Correlation and contribution values of all individual variables to the MFA´s first (**D**) and second (**E**) dimensions.

**Supplementary Table S1:** Number of individuals for each ESP and genomic ancestry categories for each country. The individuals are assigned to a given category (African (AF), Native American (NA), European (EU), Mestizo (ME) when one of them is equal or greater than 60. Otherwise, they are grouped into the ME sub-sample.

| Country | Group | ESP | Genomic ancestry | Subtotal |
| --- | --- | --- | --- | --- |
| Brazil | AF | 107 | 3 |  |
|  | AM | 59 | 6 |  |
|  | EU | 728 | 623 |  |
|  | ME | 640 | 901 |  |
|  |  |  |  | 1533 |
| Chile | AM | 339 | 211 |  |
|  | EU | 348 | 258 |  |
|  | ME | 816 | 1035 |  |
|  |  |  |  | 1504 |
| Colombia | AF | 56 | 6 |  |
|  | AM | 185 | 16 |  |
|  | EU | 143 | 495 |  |
|  | ME | 618 | 485 |  |
|  |  |  |  | 1002 |
| Mexico | AM | 494 | 609 |  |
|  | EU | 162 | 173 |  |
|  | ME | 953 | 827 |  |
|  |  |  |  | 1609 |
| Peru | AM | 131 | 250 |  |
|  | EU | 45 | 29 |  |
|  | ME | 270 | 167 |  |
|  |  |  |  | 446 |
| All Countries | AF | 163 | 9 |  |
|  | AM | 1208 | 1091 |  |
|  | EU | 1427 | 1578 |  |
|  | ME | 3296 | 3416 |  |
| Total |  |  |  | 6094 |

**Supplementary Table S2:** Wilcoxon signed-rank and Monte Carlo test between ESP and genomic ancestry for each country and ancestry in order to compare differences across age ranks. z: statistic value, P-value: <0,05 shown in bold italic, P-value permut: <0,05 shown in bold italic.

| Country | Birth decade | Native American ancestry | | African ancestry | | European ancestry | |
| --- | --- | --- | --- | --- | --- | --- | --- |
| Brazil | >1960 | Z | -0.957 | Z | -0.698 | Z | -3.663 |
|  |  | P-value | 0.339 | P-value | 0.485 | P-value | ***0.000*** |
|  |  | P-value permut. | 0.355 | P-value permut. | 0.498 | P-value permut. | ***0.000*** |
|  | 1970 | Z | -3.045 | Z | -2.293 | Z | -6.802 |
|  |  | P-value | ***0.002*** | P-value | ***0.022*** | P-value | ***0.000*** |
|  |  | P-value permut. | ***0.004*** | P-value permut. | ***0.022*** | P-value permut. | ***0.000*** |
|  | 1980 | Z | -6.616 | Z | -6.864 | Z | -14.224 |
|  |  | P-value | ***0.000*** | P-value | ***0.000*** | P-value | ***0.000*** |
|  |  | P-value permut. | ***0.000*** | P-value permut. | ***0.000*** | P-value permut. | ***0.000*** |
|  | 1990 | Z | -3.598 | Z | -4.668 | Z | -8.033 |
|  |  | P-value | ***0.000*** | P-value | ***0.000*** | P-value | ***0.000*** |
|  |  | P-value permut. | ***0.000*** | P-value permut. | ***0.000*** | P-value permut. | ***0.000*** |
| Chile | >1960 | Z | -2.157 | Z | -1.047 | Z | -1.243 |
|  |  | P-value | ***0.031*** | P-value | 0.295 | P-value | 0.214 |
|  |  | P-value permut. | ***0.029*** | P-value permut. | 0.300 | P-value permut. | 0.219 |
|  | 1970 | Z | -5.822 | Z | -2.612 | Z | -6.589 |
|  |  | P-value | ***0.000*** | P-value | ***0.009*** | P-value | ***0.000*** |
|  |  | P-value permut. | ***0.000*** | P-value permut. | ***0.010*** | P-value permut. | ***0.000*** |
|  | 1980 | Z | -11.971 | Z | -5.195 | Z | -13.391 |
|  |  | P-value | ***0.000*** | P-value | ***0.000*** | P-value | ***0.000*** |
|  |  | P-value permut. | ***0.000*** | P-value permut. | ***0.000*** | P-value permut. | ***0.000*** |
|  | 1990 | Z | -7.119 | Z | -0.132 | Z | -6.454 |
|  |  | P-value | ***0.000*** | P-value | 0.895 | P-value | ***0.000*** |
|  |  | P-value permut. | ***0.000*** | P-value permut. | 0.893 | P-value permut. | ***0.000*** |
| Colombia | >1960 | Z | -0.542 | Z | -0.944 | Z | -2.023 |
|  |  | P-value | 0.588 | P-value | 0.345 | P-value | ***0.043*** |
|  |  | P-value permut. | 0.693 | P-value permut. | 0.430 | P-value permut. | 0.065 |
|  | 1970 | Z | -2.646 | Z | -1.618 | Z | -7.619 |
|  |  | P-value | ***0.008*** | P-value | 0.106 | P-value | ***0.000*** |
|  |  | P-value permut. | ***0.009*** | P-value permut. | 0.109 | P-value permut. | ***0.000*** |
|  | 1980 | Z | -6.179 | Z | -6.126 | Z | -18.439 |
|  |  | P-value | ***0.000*** | P-value | ***0.000*** | P-value | ***0.000*** |
|  |  | P-value permut. | ***0.000*** | P-value permut. | ***0.000*** | P-value permut. | ***0.000*** |
|  | 1990 | Z | -1.753 | Z | -3.932 | Z | -10.851 |
|  |  | P-value | 0.080 | P-value | ***0.000*** | P-value | ***0.000*** |
|  |  | P-value permut. | 0.081 | P-value permut. | ***0.000*** | P-value permut. | ***0.000*** |
| Mexico | >1960 | Z | -0.486 | Z | -1.714 | Z | -1.842 |
|  |  | P-value | 0.627 | P-value | 0.086 | P-value | 0.066 |
|  |  | P-value permut. | 0.641 | P-value permut. | 0.084 | P-value permut. | 0.068 |
|  | 1970 | Z | -2.927 | Z | -0.286 | Z | -5.615 |
|  |  | P-value | ***0.003*** | P-value | 0.775 | P-value | ***0.000*** |
|  |  | P-value permut. | ***0.003*** | P-value permut. | 0.778 | P-value permut. | ***0.000*** |
|  | 1980 | Z | -8.420 | Z | -2.201 | Z | -5.745 |
|  |  | P-value | ***0.000*** | P-value | ***0.028*** | P-value | ***0.000*** |
|  |  | P-value permut. | ***0.000*** | P-value permut. | ***0.025*** | P-value permut. | ***0.000*** |
|  | 1990 | Z | -8.068 | Z | -1.123 | Z | -1.286 |
|  |  | P-value | ***0.000*** | P-value | 0.261 | P-value | 0.198 |
|  |  | P-value permut. | ***0.000*** | P-value permut. | 0.260 | P-value permut. | 0.206 |
| Peru | >1960 | Z | -2.275 | Z | -1.956 | Z | -1.726 |
|  |  | P-value | ***0.023*** | P-value | 0.050 | P-value | 0.084 |
|  |  | P-value permut. | ***0.022*** | P-value permut. | 0.051 | P-value permut. | 0.093 |
|  | 1970 | Z | -2.313 | Z | -2.109 | Z | -0.206 |
|  |  | P-value | ***0.021*** | P-value | ***0.035*** | P-value | 0.837 |
|  |  | P-value permut. | ***0.018*** | P-value permut. | ***0.031*** | P-value permut. | 0.842 |
|  | 1980 | Z | -9.418 | Z | -1.494 | Z | -1.336 |
|  |  | P-value | ***0.000*** | P-value | 0.135 | P-value | 0.182 |
|  |  | P-value permut. | ***0.000*** | P-value permut. | 0.133 | P-value permut. | 0.180 |
|  | 1990 | Z | -8.744 | Z | -2.303 | Z | -0.252 |
|  |  | P-value | ***0.000*** | P-value | ***0.021*** | P-value | 0.801 |
|  |  | P-value permut. | ***0.000*** | P-value permut. | ***0.021*** | P-value permut. | 0.805 |

**Supplementary Table S3:** Dataset used in the MFA analyses.

Self-perception groups: AF: mostly African descendants, AM: mostly Amerindian descendants, EU: mostly European descendants, and ME: mostly mestizos (number of individuals falling into each category is shown in parentheses). The variables in the dataset are quantitative (e.g. melanin values, wealth index, etc.), qualitative/categorical (eye color, baldness, hair color, graying, hair shape) or socio-cultural (wealth, education).

|  |  | **quantitative phenotypic traits** | | | **qualitative phenotypic traits** | | | | | | | | | | | | | | | | | | | | | | | **Sociocultural Variables** | | | | | |
| --- | --- | --- | --- | --- | --- | --- | --- | --- | --- | --- | --- | --- | --- | --- | --- | --- | --- | --- | --- | --- | --- | --- | --- | --- | --- | --- | --- | --- | --- | --- | --- | --- | --- |
|  |  |  | | | Eye Color | | | | | Baldness | | | | | Hair Color | | | | Graying | | | | | Hair Shape | | | | Wealth | | | Education | | |
|  |  | Weight | Stature | Melanin | blue/grey | green | honey | light brown | darkbrown/black | no hair loss | frontal bald | mild vertex bald | moderate vertex bald | severe vertex bald | red/reddish | blond | darkblond/lightbrown | brown/black | no greying | predom no-greying | 50 % greying | predom greying | totally white hair | straight | wavy | curly | afro | Wealth1 (low) | Wealth2 (medium) | Wealth3 (high) | Ed1 (low) | Ed2 (medium) | Ed3 (high) |
|  |  | **Weight** | **Stature** | **Melanin** | **Ey1** | **Ey2** | **Ey3** | **Ey4** | **Ey5** | **B1** | **B2** | **B3** | **B4** | **B5** | **HC1** | **HC2** | **HC3** | **HC4** | **G1** | **G2** | **G3** | **G4** | **G5** | **HS1** | **HS2** | **HS3** | **HS4** | **W1** | **W2** | **W3** | **Ed1** | **Ed2** | **Ed3** |
| **Brazil** | **AF** (108) | 66.99 | 165.48 | 44.69 | 3 | 3 | 2 | 12 | 85 | 101 | 3 | 2 | 0 | 0 | 1 | 1 | 16 | 87 | 90 | 17 | 0 | 0 | 0 | 9 | 25 | 47 | 25 | 39 | 27 | 28 | 12 | 55 | 36 |
|  | **AM** (59) | 67.33 | 162.23 | 37.45 | 2 | 3 | 0 | 5 | 45 | 56 | 3 | 0 | 0 | 0 | 0 | 1 | 7 | 48 | 39 | 15 | 3 | 2 | 0 | 27 | 22 | 9 | 1 | 22 | 20 | 11 | 12 | 31 | 13 |
|  | **EU** (729) | 68.27 | 167.89 | 30.46 | 96 | 160 | 41 | 173 | 255 | 641 | 65 | 10 | 9 | 2 | 14 | 86 | 323 | 303 | 514 | 136 | 50 | 22 | 6 | 387 | 246 | 93 | 2 | 184 | 194 | 297 | 92 | 249 | 385 |
|  | **ME** (640) | 66.48 | 165.88 | 34.82 | 24 | 51 | 19 | 107 | 434 | 593 | 29 | 10 | 5 | 2 | 2 | 16 | 160 | 458 | 506 | 101 | 17 | 13 | 1 | 204 | 266 | 150 | 17 | 168 | 193 | 239 | 51 | 287 | 299 |
|  |  |  |  |  |  |  |  |  |  |  |  |  |  |  |  |  |  |  |  |  |  |  |  |  |  |  |  |  |  |  |  |  |  |
| **Chile** | **AM** (339) | 69.38 | 164.05 | 38.12 | 0 | 15 | 5 | 20 | 295 | 302 | 24 | 10 | 3 | 0 | 2 | 2 | 17 | 315 | 289 | 34 | 7 | 8 | 1 | 221 | 102 | 12 | 4 | 111 | 114 | 108 | 16 | 114 | 208 |
|  | **EU** (348) | 74.68 | 169.43 | 32.87 | 23 | 51 | 26 | 44 | 202 | 278 | 51 | 10 | 9 | 0 | 5 | 25 | 82 | 233 | 264 | 44 | 28 | 8 | 4 | 186 | 130 | 18 | 14 | 102 | 94 | 142 | 15 | 172 | 161 |
|  | **ME** (816) | 73.31 | 168.03 | 35.98 | 16 | 49 | 40 | 57 | 650 | 673 | 101 | 30 | 12 | 0 | 8 | 12 | 92 | 701 | 674 | 77 | 38 | 24 | 3 | 470 | 275 | 47 | 23 | 255 | 244 | 303 | 27 | 402 | 386 |
|  |  |  |  |  |  |  |  |  |  |  |  |  |  |  |  |  |  |  |  |  |  |  |  |  |  |  |  |  |  |  |  |  |  |
| **Colombia** | **AF** (56) | 60.56 | 162.75 | 43.97 | 1 | 0 | 4 | 8 | 43 | 53 | 1 | 1 | 1 | 0 | 1 | 1 | 6 | 48 | 52 | 3 | 1 | 0 | 0 | 7 | 15 | 21 | 13 | 24 | 18 | 14 | 7 | 37 | 12 |
|  | **AM** (185) | 62.85 | 164.04 | 36 | 2 | 8 | 18 | 26 | 131 | 175 | 3 | 4 | 3 | 0 | 0 | 2 | 25 | 158 | 172 | 13 | 0 | 0 | 0 | 76 | 79 | 29 | 1 | 62 | 59 | 62 | 15 | 126 | 44 |
|  | **EU** (143) | 68.93 | 168.97 | 33.23 | 2 | 15 | 23 | 32 | 71 | 128 | 6 | 6 | 1 | 2 | 0 | 6 | 33 | 104 | 123 | 17 | 2 | 1 | 0 | 52 | 60 | 31 | 0 | 34 | 40 | 68 | 16 | 71 | 56 |
|  | **ME** (619) | 62.58 | 164.66 | 35.55 | 5 | 38 | 70 | 96 | 409 | 594 | 5 | 10 | 8 | 1 | 4 | 12 | 80 | 522 | 571 | 45 | 0 | 2 | 0 | 228 | 247 | 132 | 11 | 179 | 187 | 246 | 73 | 405 | 140 |
|  |  |  |  |  |  |  |  |  |  |  |  |  |  |  |  |  |  |  |  |  |  |  |  |  |  |  |  |  |  |  |  |  |  |
| **Mexico** | **AM** (505) | 66.28 | 161.4 | 37.8 | 0 | 10 | 8 | 61 | 415 | 433 | 49 | 6 | 3 | 2 | 1 | 1 | 55 | 436 | 334 | 101 | 37 | 20 | 2 | 275 | 182 | 34 | 3 | 151 | 165 | 172 | 144 | 158 | 180 |
|  | **EU** (163) | 70.16 | 167.25 | 32.61 | 6 | 22 | 11 | 51 | 72 | 119 | 33 | 6 | 3 | 1 | 1 | 11 | 56 | 94 | 95 | 36 | 23 | 8 | 0 | 71 | 69 | 22 | 0 | 42 | 44 | 76 | 28 | 53 | 78 |
|  | **ME** (965) | 66.7 | 163.71 | 35.95 | 9 | 39 | 27 | 216 | 662 | 822 | 98 | 22 | 7 | 3 | 2 | 14 | 167 | 770 | 694 | 196 | 36 | 23 | 2 | 390 | 419 | 135 | 4 | 284 | 275 | 391 | 283 | 263 | 385 |
|  |  |  |  |  |  |  |  |  |  |  |  |  |  |  |  |  |  |  |  |  |  |  |  |  |  |  |  |  |  |  |  |  |  |
|  |  |  |  |  |  |  |  |  |  |  |  |  |  |  |  |  |  |  |  |  |  |  |  |  |  |  |  |  |  |  |  |  |  |
| **Peru** | **AM** (132) | 60.75 | 161.29 | 37.89 | 0 | 2 | 2 | 8 | 119 | 119 | 3 | 8 | 0 | 1 | 0 | 0 | 8 | 123 | 116 | 11 | 3 | 1 | 0 | 78 | 39 | 14 | 0 | 41 | 33 | 45 | 7 | 95 | 29 |
|  | **EU** (45) | 65.37 | 164.56 | 33.75 | 2 | 4 | 3 | 6 | 30 | 36 | 3 | 5 | 1 | 0 | 0 | 1 | 12 | 32 | 42 | 3 | 0 | 0 | 0 | 21 | 19 | 5 | 0 | 5 | 7 | 29 | 2 | 37 | 6 |
|  | **ME** (271) | 64.76 | 164.25 | 36.99 | 0 | 6 | 6 | 21 | 237 | 223 | 12 | 32 | 3 | 0 | 0 | 2 | 22 | 246 | 246 | 15 | 6 | 3 | 0 | 102 | 122 | 43 | 3 | 60 | 60 | 121 | 9 | 203 | 56 |
|  |  |  |  |  |  |  |  |  |  |  |  |  |  |  |  |  |  |  |  |  |  |  |  |  |  |  |  |  |  |  |  |  |  |
| **All Countries** | **AF** (164) | 64.79 | 164.55 | 44.45 | 4 | 3 | 5 | 20 | 128 | 154 | 4 | 3 | 1 | 0 | 2 | 2 | 22 | 135 | 142 | 20 | 1 | 0 | 0 | 16 | 41 | 68 | 38 | 63 | 45 | 43 | 19 | 93 | 49 |
|  | **AM** (1220) | 66.07 | 162.58 | 37.61 | 4 | 38 | 33 | 120 | 1013 | 1085 | 82 | 28 | 9 | 3 | 3 | 6 | 112 | 1086 | 950 | 174 | 50 | 31 | 3 | 677 | 424 | 98 | 9 | 387 | 392 | 399 | 194 | 525 | 476 |
|  | **EU** (1428) | 70.03 | 168.2 | 31.68 | 129 | 252 | 104 | 308 | 632 | 1202 | 158 | 38 | 23 | 5 | 20 | 129 | 508 | 770 | 1038 | 237 | 103 | 39 | 10 | 717 | 524 | 169 | 16 | 367 | 380 | 613 | 153 | 582 | 686 |
|  | **ME** (3311) | 67.36 | 165.42 | 35.75 | 54 | 183 | 162 | 497 | 2399 | 2905 | 245 | 104 | 35 | 6 | 16 | 56 | 521 | 2703 | 2691 | 434 | 97 | 65 | 6 | 1394 | 1331 | 508 | 58 | 946 | 959 | 1300 | 443 | 1561 | 1267 |
